# Flow-diverter stents in intracranial aneurysm treatment: impact on covered cerebral artery branches

**DOI:** 10.1097/JS9.0000000000000762

**Published:** 2023-10-17

**Authors:** Junyu Liu, Fang Cao, Nibu Zhenmei, Yuxin Guo, Yifeng Li, Dun Yuan, Weixi Jiang, Junxia Yan

**Affiliations:** a Department of Neurosurgery, XiangYa Hospital; b Hunan Provincial Key Laboratory of Clinical Epidemiology; c Department of Epidemiology and Health Statistics, XiangYa School of Public Health, Central South University, Changsha, People’s Republic of China; d Department of Pharmacology, Kyoto University Graduate School of Medicine, Kyoto, Japan

**Keywords:** branch, cross-sectional study, flow diverter, intracranial aneurysm, meta-analysis, outcome

## Abstract

**Objective:**

Flow diverter stents (FDSs) have attracted interest for intracranial aneurysm (IA) treatment; however, occlusion of side branches and related complications have been reported. This study aimed to investigate the effects of FDSs in IA management when different branches of intracranial arteries are covered.

**Materials and methods:**

A cross-sectional study was conducted using PUBMED, Embase, Web of Science, and Cochrane databases to include randomized or nonrandomized comparative-designed studies from January 2000 to August 2022 which reported outcomes of occlusion/narrowing of branches after IA treatment using FDSs. The PRISMA guidelines were used for our report. A random-effects meta-analysis was conducted to pool the outcomes, which included incidence rates of occlusion/narrowing of FDS-covered branches, branch occlusion-related symptoms, obliteration of IAs, and ideal clinical outcomes (modified Rankin Scale score ≤2).

**Results:**

The authors identified 57 studies involving 3789 patients with IA managed by FDSs covering different branches. During the median imaging follow-up at 12 months, the IA obliteration rate was satisfactory (>70%) when covering the ophthalmic artery (OA), posterior communicating artery (PComA), anterior choroidal artery (AChoA) or anterior cerebral artery (ACA), but not the middle cerebral artery-M2 segment (MCA-M2; 69.5%; 95% CI: 60.8–77.5%) and posterior inferior cerebellar artery (PICA; 59.1%, 13/22). The overall ideal clinical outcome was observed in 97.4% of patients (95% CI: 95.5–98.9%). Higher rates of occlusion/narrowing of branches were identified when FDSs covered the ACA (66.6%; 95% CI: 45.1–85.3%), PComA (44.3%; 95% CI: 34.2–54.6%), or MCA-M2 (39.2%; 95% CI: 24.5–54.7%); the risks were lower when covering the OA (11.8%; 95% CI: 8.8–15.1%), PICA (6.8%; 95% CI: 1.5–14.5%), and AchoA (0.5%; 95% CI: 0.0–2.9%). The risk of branch occlusion-related complications was low (incidence rate <5%) for each of the six evaluated branches.

**Conclusions:**

Acceptable outcomes were identified following treatment of IAs when FDSs were placed across each of the six studied cerebral arteries. Treatment decisions regarding FDS placement across branch arteries should be made with the risk of complications from branch occlusion in mind.

## Introduction

Highlights

- Among the 57 studies, 3789 patients with IA were involved who were treated by FDSs. The overall obliteration rate was 77.5%, (95% CI: 74.0–80.9%) and 97.4% of patients (95% CI: 95.5–98.9%) had a modified Rankin Score ≤2 at the last follow-up (median, 12 months).
- The pooled rates of branch occlusion-related complications were less than 5% in the evaluated cerebral branches.
- Acceptable safety and efficacy were identified in the treatment of IA when FDSs were placed across branches.

The rupture of intracranial aneurysms (IAs) results in poor outcomes for ~35% of patients with subarachnoid hemorrhage (SAH)^1^. Endovascular treatment has become a prime choice for IA treatment and the prevention of IA rupture. Flow diverter stents (FDSs), a customized tool, were initially designed for the management of large and wide-necked IA located in the internal carotid artery (ICA), and displayed immediate and long-term efficacy and safety^2^. As the technique matures, there have been several new attempts to apply FDS to treat IAs in the posterior circulation and other arteries beyond the circle of Willis^3^. However, when placed in a small-diameter artery, FDSs can induce stenosis or occlusion of the parent artery because of their higher metal mesh density compared to conventional stents. The induced changes in hemodynamics can reduce blood flow in FDS-covered vessels, accelerate thrombus formation within the branches, and promote IA occlusion, increasing the risk of intracranial thrombosis, and becoming an important cause of severe complications and mortality. Reliable evidence regarding the risks of occlusion in different branches, related complications, and their potential mechanisms is needed to demonstrate the safety and efficacy of the procedure, and whether the indications for FDSs could be extended to IAs beyond the ICA in future clinical applications. Thus, this study aims to comprehensively summarize the existing evidence and assess the patency of intracranial arterial branches and related complications when they are covered by FDSs in the treatment of IAs.

## Materials and methods

This study adhered to strengthening the reporting of cohort studies in surgery (STROCSS) criteria^4^ (Supplemental Digital Content 1, http://links.lww.com/JS9/B225), PRISMA 2020 guidelines^5^ and AMSTAR guidelines^6^ and was registered with PROSPERO (CRD42022350339). Three authors (J.Y.L., F.C., N.B.Z.M.) independently performed record screening and data collection, while two authors (J.Y.L. and F.C.) conducted the risk-of-bias assessment. Subsequently, another author (Y.X.G.) reviewed the information for accuracy. Disagreements were discussed and resolved with J.X.Y. to reach a consensus.

### Search strategy

We conducted a search in four databases—(PUBMED, Embase, Web of Science, and Cochrane databases)—without language restrictions, up to August 2022. The keywords included ʻintracranial aneurysmʼ, ʻcerebral aneurysmʼ, ʻflow diverterʼ, ʻpipelineʼ, ʻsurpassʼ, ʻsilk,” ʻfredʼ, ʻtubridgeʼ, ʻderivoʼ, ʻp64ʼ, and ʻp48ʼ. Appendix 1 (Supplemental Digital Content 1, http://links.lww.com/JS9/B225) provides the complete search strategies.

### Study selection

The studies included had a randomized or nonrandomized comparative design and had enrolled a minimum of 10 patients with IA. Studies needed to report data on occlusion/narrowing rates of branches and involve patients with IAs that had been treated with FDSs, regardless of the IA’s location. We excluded reviews, case reports, animal studies, conference abstracts, and case series with fewer than 10 patients.

### Data extraction

The characteristics of each included study were extracted according to a prespecified procedure. The outcomes of efficacy were identified as obliteration of IAs and ideal clinical outcomes (using the modified Rankin Scale^7^, less than 2, at the last clinical follow-up), and the outcomes of complications included occlusion/narrowing of FDS-covered branches and branch occlusion-related symptoms. Patients experiencing a symptomatic acute stroke event were included as having a branch occlusion-related symptom, whether the condition was temporary or permanent. Most of these patients were evaluated through angiography when clinical symptoms appeared. For nonsymptomatic patients, branch occlusion/narrowing was identified in routine imaging follow-up. Details were described in Supplemental Methods (Supplemental Digital Content 2, http://links.lww.com/JS9/B226).

### Risk-of-bias assessment

The risk-of-bias in each study was rated using the Quality in Prognosis Studies (QUIPS) tool^8^ developed for prognosis studies. Six domains were considered to evaluate the validity and bias in prognostic factors: study participation, study attrition, prognostic factor measurement, confounding measurement and accounting, outcome measurement, and analysis and reporting (details in Appendix 2, Supplemental Digital Content 1, http://links.lww.com/JS9/B225). We categorized the overall quality of the studies based on the risk-of-bias in each domain: studies with no domain at high risk were considered ‘high quality,’ those with one domain at high risk were ‘moderate quality,’ and those with more than one domain at high risk were deemed ‘low quality.’

### Statistical analysis

Statistical analyses were performed using the R meta package and software (R version 4.1.2; R Foundation for Statistical Computing). Random-effects meta-analyses of single proportions were conducted to obtain summary estimates of the incidence rates of outcomes for conservative results. The incidence rates of branch occlusion-related symptoms were estimated according to the risk of complications among the included patients or the risk caused by occluded/narrowed branches. Considering the presence of the rates of outcomes at 0% in several studies, the 95% CIs were calculated using the score method, and the variance-stabilizing Freeman-Tukey transformation was applied to avoid excluding those studies from evidence synthesis. Cochran’s *Q* test, tau (τ), and Higgins’ *I*^2^ statistics were used to evaluate heterogeneity. Subgroup analysis was conducted according to the published year of studies, location of the IA, follow-up period, location of medical centers, sample size, and covered branches [six branches of the ophthalmic artery (OA), posterior communicating artery (PComA), anterior choroidal artery (AChoA), anterior cerebral artery (ACA), middle cerebral artery-M2 segment (MCA-M2) and posterior inferior cerebellar artery (PICA)]. We performed a sensitivity analysis in two ways: first, by excluding each study individually to assess the remaining studies, and second, by including only those studies with a sample size of greater than or equal to 20. Funnel plot asymmetry was tested using Egger’s test to investigate the association between the sample size and outcomes when at least 10 studies were included. Statistical significance was set at a two-sided *P*-value of <0.05. When *P*<0.05 in Egger’s test, the trim-and-fill method was used to reduce publication bias.

## Results

A total of 57 studies^9–65^, encompassing 4647 records, involved 3789 patients with IA who were treated using FDSs. These studies employed a nonrandomized comparative design; the selection process is shown in Figure S1 (Supplemental Digital Content 2, http://links.lww.com/JS9/B226). The median total sample size was 28 patients (interquartile range: 19.5–58.5), the median age of participants was 54 years (52–58), and the median percentage of men was 25% (16–36%). The median imaging follow-up period was 12 months (7.9–15.2). In 75.4% (43/57) of the studies, outcomes were assessed using digital subtraction angiography as the sole imaging modality. The detailed characteristics of each study are listed in Table 1 and S2.

**Table 1:** Summary of included studies and baseline characteristics.

References	Year	Country	Medical Center (Period)	Study Design	No. of Patients	Male, n(%)	Age, mean (SD/range)	Location of IA	Flow Diverter Stents	Covered branch	Preoperative Antiplatelet Treatment^a^	Postoperative Antiplatelet Treatment^a^	Follow-up Rate (%)	Average Imaging Follow-up (month)	Imaging modality	Occlusion/narrowing of Branch (%)	Branch occlusion-related symptom (per patient)	Obliteration Rate (%)	Ideal Clinical Outcome (%)
Szikora *et al.* ^9^	2010	Hungary	Singlecenter (2007–2008)	case series	18	NA	NA	ICA-clinoid/ OA/PComA segment, BA	PED	–	Aspirin 100 mg daily and clopidogrel 75 mg daily for at least 2 day before surgery	Aspirin 100 mg daily for lifelong and clopidogrel 75 mg daily for 6 weeks	17/18 (94.4)	6.0	MRA, CTA	3/28 (10.7)	1/28 (3.6)	16/17 (94.1)	NR
Pillips *et al.* ^10^	2012	Australia	Multicenter (2009–2011)	cohort study	32	NA	NA	Posterior circulation	PED	–	Aspirin and clopidogrel for 5–7 days (details NR)	Dual antiplatelet therapy (details NR)	28/32 (87.5)	21.0	DSA, MRA, CTA	3/32 (9.4)	3/32 (9.4)	24/28 (85.7)	32/32 (100.0)
Pistocchi *et al.* ^11^	2012	France	Singlecenter (2008–2011)	case series	26	9 (34.6)	48 (10–77)	Anterior circulation	PED, SED	–	UIA: Iaspirin 250 mg intravenously; RIA: abciximab 0.25 mg/kg intravenously	aspirin 250 mg daily and clopidogrel 75–150 mg daily for 6 months	20/26 (76.9)	13.0	DSA	13/21 (61.9)	0/20 (0.0)	19/23 (82.6)	20/21 (95.2)
Briganti *et al.* ^12^	2014	Italy	Multicenter (2010–2013)	case series	14	4 (28.6)	59 (39–71)	MCA-M1/bifucation	PED	MCA-M2	clopidogrel 75 mg daily for 5 days and aspirin 150 mg	aspirin 100 mg daily for lifelong, and clopidogrel 75 mg daily until aneurysm occlusion	14/14 (100.0)	≧6.0	DSA, CTA	9/13 (69.2)	1/12 (16.7)	12/15 (80.0)	NR
Brinjikji *et al.* ^13^	2014	USA	Singlecenter (2011–2012)	case series	11	2 (18.2)	52 (11.6)	ICA-PComA segment or ICA bifurcation	PED	PComA	Aspirin and clopidogrel (details NR)	Aspirin for lifelong and clopidogrel for 3 months (details NR)	11/11 (100.0)	12.6	DSA	5/11 (45.5)	0/11 (0.0)	9/11 (81.8)	NR
Martinez- Galdamez *et al.* ^14^	2014	Spain, USA	Multicenter (NR)	case series	25	NA	NA	distal arteries	PED	–	Aspirin 100–325 mg daily and clopidogrel 75 mg daily/ticagrelor (details NR) for 5 days	Dual antiplatelet therapy for follow-up	22/25 (88.0)	6.0	DSA	3/14 (21.4)	0/17 (0.0)	14/22 (63.6)	24/25 (96.0)
Moon *et al.* ^15^	2014	USA	Singlecenter (2011–2012)	cohort study	30	0 (0.0)	56 (NA)	ICA-OA segment	PED	OA	Aspirin and clopidogrel (details NR)	Dual antiplatelet therapy for at 6–12 months, then aspirin treatment alone (details NR)	29/30 (93.3)	≧6.0	DSA	1/29 (3.4)	0/29 (0.0)	30/38 (78.9)	NR
Saleme *et al.* ^16^	2014	France	Singlecenter (2011–2013)	case series	32	12 (37.5)	54 (11.8)	bifucation of AComA, ACA, MCA, ICA-terminal	PED	–	UIA: clopidogrel 75 mg daily for 6 days; RIA: 0.25 mg/kg abciximab intravenously during surgery, then 0.125 mg/ (kg·min) for 12 hours after surgery	aspirin for 12 months, and clopidogrel for 6 months (details NR)	32/32 (100.0)	≧18.0	DSA	27/37 (73.0)	5/37 (13.5)	36/37 (97.3)	31/32 (96.9)
Brinjikji *et al.* ^17^	2015	USA	Singlecenter (2010–2013)	case series	15	3 (20.0)	56 (14.4)	ICA-clinoid/ OA/PComA segment, BA	PED	AchoA	aspirin and clopidogrel (details NR)	aspirin for lifelong and clopidogrel for 3 months (details NR)	14/15 (93.3)	12.0	MRA, CTA	1/14 (7.1)	0/15 (0.0)	7/14 (50.0)	14/15 (93.3)
Chalouhi *et al.* ^18^	2015	USA	Singlecenter ( -2013)	cohort study	95	10 (10.5)	53 (13.0)	ICA-clinoid/ OA/PComA segment, MCA	PED	OA	aspirin 81 mg daily and clopidogrel 75 mg daily for 10 days	dual antiplatelet therapy (details NR)	95/95 (100.0)	7.5	DSA	10/95 (10.5)	1/95 (1.1)	73/95 (76.8)	NR
Durst *et al.* ^19^	2015	USA	Singlecenter (NR)	case-control study	19	3 (16.7)	53 (3.1)	ICA-OA segment	PED	OA	NR	NR	19/19 (100.0)	12.0	DSA, MRA, CTA	5/19 (26.3)	2/19 (10.5)	14/19 (73.7)	17/19 (89.5)
Gascou *et al.* ^20^	2015	France	Multicenter (2009-2012)	Cohort study	59	13 (22.0)	54 (NA)	ICA, ACA, MCA, posterior circulation arteries	PED	–	Clopidogrel 300 mg the day before surgery	Aspirin 75 mg/160 mg daily for lifelong, clopidogrel 75 mg daily for 6 months	58/59 (98.3)	≧12.0	DSA, MRA	13/68 (19.1)	8/58 (13.8)	NR	51/58 (87.9)
Neki *et al.* ^21^	2015	France	Singlecenter (2011–2013)	case series	20	6 (30.0)	58 (22-74)	ICA-clinoid/ OA/PComA segment or ICA bifurcation	PED, SFD, FRED, Surpass	AchoA	Aspirin 160 mg daily and clopidogrel 75 mg daily for 7 days	Aspirin for 12 months and clopidogrel for 3 months (details NR)	17/20 (85.0)	9.8	DSA	0/17 (0.0)	0/17 (0.0)	NR	NR
Raz *et al.* ^22^	2015	USA	NA (2008–2013)	cohort study	28	7 (25.0)	58 (13.6)	ICA	PED	AchoA	NR	aspirin 325 mg daily and clopidogrel 75 mg daily for at least 6 months	28/28 (100.0)	15.1	DSA	3/28 (10.7)	1/28 (3.6)	25/28 (89.3)	27/28 (96.4)
Rouchaud *et al.* ^23^	2015	France	Singlecenter (2009–2013)	cohort study	28	9 (32.1)	NA (19–81)	ICA-OA segment	PED	OA	aspirin 160 mg daily and clopidogrel 75 mg daily for 7 days, aspirin 250 mg intravenously before surgery	aspirin 160 mg daily and clopidogrel 75 mg daily for 3 months	28/28 (100.0)	≧12.0	DSA	4/28 (14.3)	4/28 (14.3)	22/28 (78.6)	NR
Vedantam *et al.* ^24^	2015	USA	Singlecenter (2011–2013)	cohort study	64	6 (12.2)	56 (1.8)	ICA-cavernous/ clinoid/ OA/PComA segment	PED	–	aspirin and clopidogrel for at least 10 days (details NR)	aspirin daily for lifelong, and clopidogrel daily for 6 months (details NR)	49/64 (76.6)	12.8	DSA	6/74 (8.1)	6/74 (8.1)	NR	NR
Bhogal *et al.* ^25^	2016	Germany	Singlecenter (2009–2016)	case series	26	11 (42.3)	59 (27–77)	ACA	PED, p64 FD	–	aspirin 100 mg daily and clopidogrel 75 mg daily/ticagrelor 90 mg twice daily (details NR)	aspirin 100 mg daily for lifelong and clopidogrel 75 mg daily/ticagrelor 90 mg twice daily for 12 months	20/26 (76.9)	3.1	DSA	0/20 (0.0)	0/20 (0.0)	16/20 (80.0)	NR
Burrows *et al.* ^26^	2016	USA	Singlecenter (2009–2015)	Cohort study	44	3 (6.3)	52 (14.0)	ICA-OA segment	PED, Surpass	OA	aspirin and clopidogrel for not less than 5 days (details NR)	aspirin for lifelong, and clopidogrel for 3 months (detailed NR)	44/44 (100.0)	29.0	DSA	8/37 (21.6)	0/37 (0.0)	29/37 (78.4)	NR
Caroff *et al.* ^27^	2016	France	Singlecenter (2013-2014)	Case series	14	5 (26.3)	NA (40–67)	MCA bifucation	PED, SFD, FRED	MCA-M2	aspirin 160 mg daily and clopidogrel 75 mg daily for 7 days	aspirin 160 mg daily for 12 months, and clopidogrel 75 mg daily for 3 months	12/14 (85.7)	16.0	DSA	8/12 (66.7)	6/12 (50.0)	8/13 (61.5)	NR
Carvalho *et al.* ^28^	2016	France	NR (2011-2015)	Case series	17	3 (17.6)	50 (23–70)	ICA-clinoid/ OA/PComA segment	PED, SFD, FRED, Surpass	PComA	Aspirin 160 mg daily and clopidogrel 75 mg daily for 7 days	Dual antiplatelet therapy (details NR)	17/17 (100.0)	10.5	DSA	10/18 (55.6)	0/18 (0.0)	13/18 (72.2)	NR
Lin *et al.* ^29^	2016	USA	Multicenter (2011–2013)	Case series	28	10 (35.7)	52 (16.7)	distal arteries	PED	–	Aspirin 325 mg daily and clopidogrel 75 mg daily; emergent basis: aspirin 650 mg and clopidogrel 600 mg before surgery	Dual antiplatelet therapy for at least 3 months (details NR)	27/28 (96.4)	7.7	DSA	3/27 (11.1)	0/27 (0.0)	21/27 (77.8)	27/28 (96.4)
Topcuoglu *et al.* ^30^	2016	Turkey	NR (2010–2015)	Case series	23	14 (50.0)	47 (3-65)	MCA	SFD, Surpass	MCA-M2	Aspirin 300 mg daily and clopidogrel 75 mg daily for 5–10 days	Aspirin 300 mg daily for lifelong, and clopidogrel 75 mg daily for 6 months	23/23 (100.0)	10.8	DSA	5/16 (31.3)	0/17 (0.0)	16/24 (66.7)	NR
Bhogal *et al.* ^31^	2017	Germany	Singlecenter (2009–2016)	Cohort study	140	31 (21.9)	56 (13.7)	ICA-clinoid/OA/PComA segment	PED, p64 FD	–	aspirin 100 daily, clopidogrel 75 mg daily/ticagrelor 90 mg twice daily for at least 5 days	aspirin 100 mg daily for lifelong, and clopidogrel 75 mg daily/ticagrelor 90 mg twice daily for 12 months	140/140 (100.0)	22.3	DSA	49/285 (17.2)	NR	116/147(78.3)	NR
Bhogal *et al.* ^32^	2017	Germany	Singlecenter (2010-2016)	Case series	13	6 (46.2)	62 (47-74)	MCA bifucation	PED, p64 FD	MCA-M2	Aspirin 100 mg daily and clopidogrel 75 mg daily/ticagrelor 90 mg twice daily	Aspirin 100 mg daily for lifelong, and clopidogrel 75 mg daily/ticagrelor 90 mg twice daily for 12 months	12/13 (92.3)	15.8	DSA	6/12 (50.0)	0/12 (0.0)	11/12 (91.7)	NR
Daou *et al.* ^33^	2017	USA	Singlecenter (2011–2014)	Cohort study	30	6 (20.0)	54 (11.6)	ICA-PComA segment	PED	PComA	aspirin 81 mg daily and clopidogrel 75 mg daily for 10 days	antiplatelet therapy for at least 6 months	30/30 (100.0)	6.0	DSA	23/30 (76.7)	0/30 (0.0)	25/30 (83.3)	NR
Griessenauer *et al.* ^34^	2017	North & South America	Multicenter (2010–2015)	Cohort study	127	16 (12.6)	54 (11.5)	ICA-clinoid/OA segment	PED, SED	OA	UIA: aspirin 81 mg/325 mg daily and clopidogrel 75 mg daily for 14 days; RIA: aspirin 650 mg and clopidogrel 600 mg before surgery	Aspirin daily for lifelong, and clopidogrel daily for 3–6 months (details NR)	101/160 (63.1)	≧18.0	DSA	6/85 (7.1)	4/127 (3.1)	90/101 (89.1)	NR
Losif *et al.* ^35^	2017	France, Turkey	Multicenter (2010-2014)	Cohort study	58	17 (29.3)	52 (11.1)	MCA bifucation	PED, SFD, FRED	MCA-M2	Center 1. aspirin 300 mg daily and clopidogrel 75 mg/ticlopidine 600 mg daily (details NR); Center 2. UIA: clopidogrel 75 mg daily for 6 days; RIA: 0.25 mg/kg abciximab intravenously during surgery, then 0.125 mg/ (kg·min) for 12 hours after surgery	Center 1. aspirin 300 mg daily for lifelong, and clopidogrel 75 mg daily for 6 months; Center 2. aspirin for 12 months, and clopidogrel for 6 months (details NR)	58/58 (100.0)	≧12.0	DSA	15/63 (23.8)	2/63 (3.2)	45/63 (71.4)	58/58 (100.0)
Rangel- Castilla *et al.* ^36^	2017	USA	NR (2009-2014)	Cohort study	82	5 (6.1)	59 (11.7)	ICA-cavernous/ clinoid/OA/PComA segment	PED	–	Aspirin 325 mg daily and clopidogrel 75 mg daily for 5 days/aspirin 650 mg and clopidogrel 600 mg before surgery	Aspirin for 15–18 months for lifelong, and clopidogrel for at least 6 months (details NR)	82/82 (100.0)	10.0	DSA	13/127 (10.2)	0/127 (0.0)	NR	NR
Srinivasan *et al.* ^37^	2017	France	Singlecenter (2009–2015)	Case series	15	NA	NA	ICA-OA segment	PED, SFD, FRED, Surpass	OA	Aspirin 75 mg daily and clopidogrel 75 mg daily/ticagrelor 90 mg twice daily for 5 days	Dual antiplatelet therapy for 3-6 months, then aspirin only for 6–9 months (details NR)	15/15 (100.0)	12.0	DSA	20/66 (30.3)	0/18 (0.0)	15/18 (83.3)	NR
Touze *et al.* ^38^	2017	France	Singlecenter (2009–2015)	Case series	15	1 (6.7)	49 (6.1)	ICA-OA segment	PED, SFD, FRED, Surpass	OA	aspirin 75 mg daily and clopidogrel 75 mg daily/ticagrelor 90 mg twice daily for 5 days	dual antiplatelet therapy for 3–6 months, then aspirin only for 6–9 months (details NR)	15/15 (100.0)	12.0	DSA	3/16(18.8)	0/16 (0.0)	15/16 (93.7)	15/15 (100.0)
Adeeb *et al.* ^39^	2018	USA	Multicenter (2009–2016)	Cohort study	129	47 (36.4)	58 (27–82)	posterior circulation arteries	PED	AICA, PICA, VA, SCA, PCA	Aspirin 325 mg daily and clopidogrel 75 mg daily (600 mg 24 hours before surgery) for 3–14 days	Dual antiplatelet therapy for at least 3 months (details NR)	129/129 (100.0)	11.0	DSA, MRA	25/228 (11.0)	21/228 (9.2)	85/128 (66.4)	99/125(79.2)
Bhogal *et al.* ^40^	2018	Germany	Singlecenter (2009–2016)	Case series	15	4 (25.0)	58 (14-76)	MCA-M1	PED, p64 FD	LA	Aspirin 100 mg daily and clopidogrel 75 mg/ticagrelor 180 mg daily for 7 days	Aspirin for lifelong, and clopidogrel/ticagrelor for 12 months (details NR)	14/15 (93.3)	18.7	DSA	2/14 (14.3)	0/14 (0.0)	8/14 (57.1)	NR
Kuhn *et al.* ^41^	2018	USA	Multicenter (2011–2017)	Case series	56	9 (16.1)	56 (19–82)	ICA-PComA segment	PED	PComA	aspirin 81 mg daily and clopidogrel 75 mg daily for at least 5 days	aspirin for lifelong, and clopidogrel for at least 6 months (details NR)	52/56 (92.8)	NA	DSA, MRA, CTA	24/52 (46.2)	0/54 (0.0)	39/52 (75.0)	54/56 (96.4)
Liang *et al.* ^42^	2018	China	Singlecenter (2015-2016)	Case-control study	35	26 (74.3)	50 (8–62)	posterior circulation arteries	PED	–	Aspirin 100 mg daily and clopidogrel 75 mg/300 mg daily for at least 5 days	Aspirin 100 mg daily for 6 months and clopidogrel 75 mg/300 mg daily for 3 months	36/38 (94.7)	5.5	DSA	0/18 (0.0)	0/18 (0.0)	33/36 (91.7)	35/35 (100.0)
Roy *et al.* ^43^	2018	USA	Singlecenter (NR)	Cohort study	50	8 (16.0)	58 (12.2)	ICA-PComA segment	PED	PComA	dual antiplatelet therapy (details NR)	dual antiplatelet therapy for 6 months (details NR)	48/50 (96.0)	14.0	DSA, MRA	14/25 (56.0)	NR	30/48 (62.5)	NR
Wallece *et al.* ^44^	2018	USA	Multicenter (2012–2017)	Case series	14	2 (14.3)	66 (41–76)	PICA	PED	PICA	NR	NR	12/14 (85.7)	15.2	DSA	1/12 (8.3)	0/12 (0.0)	7/12 (58.3)	NR
Enriquez- Marulanda *et al.* ^45^	2019	USA	Multicenter (2013–2017)	Case series	57	8 (14.0)	61 (12.8)	ICA-PComA segment	PED	PComA	UIA: aspirin 81 mg/325 mg daily and clopidogrel 75 mg daily for 14 days; RIA: aspirin 650 mg and clopidogrel 600 mg/aspirin 81 mg and ticagrelor 180 mg before surgery	aspirin 325 mg daily for lifelong, and clopidogrel 75 mg daily/ticagrelor 90 mg twice daily/prasugrel 10 mg daily for at least 3 months	50/57 (87.7)	8.5	DSA, MRA, CTA	8/35 (22.9)	NR	42/50 (84.0)	50/53 (94.4)
Michelozzi *et al.* ^46^	2019	France	Singlecenter (2010–2017)	Case series	29	15 (51.7)	NA	distal arteries	PED, SFD, FRED	–	Double antiplatelet therapy for at least 7 days (details NR)	Aspirin for at least 15 months, and clopidogrel for 3 months (details NR)	28/29 (96.6)	21.0	DSA, MRI	23/34 (58.8)	5/29 (17.2)	22/28 (78.5)	29/29 (100.0)
Pujari *et al.* ^47^	2019	USA	Singlecenter (2011–2017)	Case series	27	8 (29.7)	52 (14.9)	ICA, MCA	PED	ACA	Dual antiplatelet therapy (details NR)	Dual antiplatelet therapy for 6 months (details NR)	27/27 (100.0)	9.2	DSA	10/27 (63.0)	0/27 (0.0)	13/27 (48.1)	NR
Raymond *et al.* ^48^	2019	USA	Singlecenter (2011–2017)	Cohort study	84	21 (25.0)	NA	–	PED	–	NR	NR	84/84 (100.0)	13.0	DSA	37/142 (26.1)	NR	NR	NR
Schob *et al.* ^49^	2019	Germany	Singlecenter (2018)	Case series	25	8 (32.0)	48 (18–70)	distal arteries	SFD	–	Aspirin 500 mg and ticagrelor 180 mg at the day before surgery	Aspirin 100 mg daily for lifelong, ticagrelor 90 mg twice daily for a year	22/25 (88.0)	2.9	DSA	12/24 (50.0)	0/24 (0.0)	17/24 (70.8)	NR
Wang *et al.* ^50^	2019	China	Singlecenter (2014-2018)	Case-control study	42	67 (85.9)	49 (12.6)	VBA	PED	AICA, PICA, VA	UIA: aspirin 100 mg daily and clopidogrel 75 mg daily for 5 days; RIA: aspirin 300 mg and clopidogrel 300 mg 2 hours before the surgery	Aspirin 100 mg daily for lifelong, clopidogrel 75 mg daily for 3 months	41/42 (97.6)	15.8	DSA	4/25 (16.0%)	1/26 (3.8)	37/41 (90.2)	40/42 (95.2)
Wu *et al.* ^51^	2019	China	Singlecenter (2015-2018)	Cohort study	126	28 (22.2)	53 (11.3)	–	PED	–	Aspirin 100 mg daily and clopidogrel 75 mg daily for 5 days	Aspirin daily for lifelong and clopidogrel daily for 6 months (details NR)	126/126 (100.0)	7.9	DSA	53/173 (30.6)	6/173 (3.5)	NR	NR
Cimflova *et al.* ^52^	2020	Turkey	Singlecenter (2013–2020)	Case series	23	8 (34.8)	NA (15-72)	MCA	PED, SFD, FRED	–	Aspirin 100–300 mg daily and clopidogrel 75 mg (loading dose 300 mg) daily for 5 days	Aspirin 100 mg daily for 15–18 months or lifelong, and clopidogrel 75 mg daily for 9–12 months/ prasugrel 10 mg (loading dose 40 mg) daily for a year	20/23 (87.0)	NA	DSA	1/19 (5.3)	0/19 (0.0)	14/20 (70.0)	NA
Hohenstatt *et al.* ^53^	2020	Italy	Singlecenter (2009–2018)	Cohort study	112	25 (22.3)	48 (18–70)	–	PED, SFD, FRED, FRED Jr, DED	–	Aspirin 300 mg daily and clopidogrel 75 mg daily for 5 days	Aspirin 300 mg daily for a year, clopidogrel 75 mg daily for 3 months	112/112 (100.0)	≧12.0	DSA	28/214 (13.1)	NR	83/119 (69.7)	NR
Cagnazzo *et al.* ^54^	2021	France	Singlecenter (2014-2020)	Case series	20	4 (20.0)	53 (12.6)	ICA bifucation	PED	ACA	Aspirin 75 mg daily and clopidogrel 75–150 mg/prasugrel 10–20 mg daily for 5 days	Aspirin 75 mg daily for 12 months, and clopidogrel 75–150 mg/prasugrel 10–20 mg daily for 6 months	20/20 (100.0)	13.0	DSA	19/20 (95.0)	0/20 (0.0)	19/20 (95.0)	NR
Diestro *et al.* ^55^	2021	Canada	Multicenter (NR)	Cohort study	54	20 (37.0)	NA (12–79)	MCA	PED	–	Dual therapy antiplatelet (details NR)	Dual antiplatelet therapy for 6 months, then for single antiplatelet therapy (details NR)	54/54 (100.0)	12.0	DSA, MRA, CTA	8/46 (17.4)	NA	25/45 (55.6)	49/52 (94.2)
Kan *et al.* ^56^	2021	USA, Europe	Multicenter (NR)	Cohort study	38	NA	64 (NA)	ICA-PComA segment	Surpass	PComA	Aspirin 75–325 mg daily and clopidogrel 75 mg daily for at least 5 days	Aspirin 75–325 mg daily for lifelong and clopidogrel 75 mg daily for 6 months	36/38 (94.7)	≧12.0	DSA	11/19 (57.9)	0/19 (0.0)	28/36 (77.8)	36/37 (97.3)
Kang *et al.* ^57^	2021	China	Multicenter (2014–2019)	Cohort study	1171	358 (30.6)	54 (11.6)	–	PED	–	Aspirin 100–300 mg daily and clopidogrel 75 mg daily/aspirin 100 mg daily and ticagrelor 90 mg twice daily (details NR)	Aspirin 100–300 mg daily and clopidogrel 75 mg daily/aspirin 100 mg daily and ticagrelor 90 mg twice daily (details NR)	858/1171 (73.2)	9.0	DSA	80/707 (11.3)	NR	787/967 (81.4)	1111/1171 (94.9)
Lee *et al.* ^58^	2021	Korea	Singlecenter (2013–2020)	Case series	12	9 (75.0)	55 (42-77)	VA	PED, FRED, Surpass	PICA	aspirin 100 mg daily and clopidogrel 75 mg daily for at least 5 days	aspirin for lifelong, and clopidogrel for 6 months (details NR)	10/12 (83.3)	6.0	DSA	0/10 (0.0)	0/10 (0.0)	6/10 (60.0)	11/12 (91.7)
Litao *et al.* ^59^	2021	USA	NR (2008–2018)	Case series	14	2 (14.3)	NA (38–82)	ICA-PComA segment	PED	PComA	Aspirin and plavix for 5–7 days (details NR)	Dual antiplatelet therapy for 6 months (details NR)	14/14 (100.0)	9.0	DSA	10/14 (71.0)	0/14 (0.0)	10/14 (71.0)	NR
Salem *et al.* ^60^	2021	USA, Europe	Multicenter (2011-2018)	Cohort study	87	60 (69.0)	58 (NA)	MCA bifucation	PED, SFD, FRED, Atlas, p48 FD, Surpass	MCA-M2	Aspirin 325 mg daily and clopidogrel 75 mg daily/ticagrelor 180 mg twice daily/prasugrel 10 mg daily for at least 7–10 days	Aspirin for lifelong and clopidogrel for at least 3 months (details NR)	78/87 (89.7)	16.3	DSA, MRA, CTA	12/78 (15.4)	2/78 (2.6)	46/77 (59.7)	60/62 (97.7)
Xu *et al.* ^61^	2021	China	Multicenter (2015-2019)	Cohort study	48	17 (35.4)	54 (13.8)	ICA bifucation	PED	ACA	Aspirin 100 mg/300 mg daily and clopidogrel 75 mg daily/ticagrelor 90 mg twice daily (details NR)	Aspirin 100 mg daily for lifelong and clopidogrel 75–150 mg daily/ticagrelor 90 mg twice daily for 6 months	48 /48 (100.0)	6.6	DSA	31/51 (64.6)	1/51 (2.0)	31/51 (64.6)	NR
Gundogmus *et al.* ^62^	2022	Turkey	Singlecenter (2012-2019)	Cohort study	138	19 (13.8)	51 (29-76)	ICA	PED, FRED	–	Aspirin 100 mg daily and clopidogrel 75 mg daily for 8 days, prasugrel 40 mg 12 hours before surgery/ticagrelor 180 mg 4h before surgery as a load in case of resistance to clopidogrel	Aspirin 100 mg daily for lifelong, and clopidogrel 75 mg daily/ticagrelor 90 mg twice daily for 6 months, or only prasugrel 40 mg daily for 6 months and then aspirin 100 mg daily for lifelong	128/138 (92.8)	33.0	DSA	20/25 (80.0)	0/25 (0.0)	148/162 (91.4)	NA
Lauzier *et al.* ^63^	2022	USA	Multicenter (2011-2020)	Case series	25	10 (40.0)	54 (16–72)	MCA	PED	–	Aspirin and clopidogrel/ticagrelor as dual antiplatelet therapy	Dual antiplatelet therapy for 6–12 months and then aspirin for lifelong (details NR)	24/25 (96.0)	30.0	DSA	7/21 (33.3)	0/21 (0.0)	17/24 (70.8)	NR
Li *et al.* ^64^	2022	China	Singlecenter (2017-2020)	Case series	21	11 (39.3)	52 (31–78)	distal arteries	PED, Turbridge FD	–	Aspirin 100 mg daily and clopidogrel 75 mg daily/ticagrelor 90 mg twice daily	Aspirin 100 mg daily for lifelong, and clopidogrel 75 mg daily/ticagrelor 90 mg twice daily for 3 months	13/21 (61.9)	10.1	DSA, MRA	3/17 (17.6)	2/19 (10.5)	NR	NR
Xu *et al.* ^65^	2022	China	Multicenter (2014–2019)	Cohort study	189	38 (20.1)	53 (9.4)	ICA-peri OA segment	PED	OA	Aspirin 100 mg/300 mg daily and clopidogrel 75 mg daily/ticagrelor 90 mg twice daily for 5 days	Aspirin 100 mg daily for lifelong, and clopidogrel 75 mg/ticagrelor 90 mg daily for 6 months	189/189 (100.0)	6.8	DSA	13/194 (6.7)	7/189 (3.7)	163/194 (84.0)	189/189 (100.0)

a antiplatelet treatment usually taken orally, except for when otherwise specified.

+, not less than the rate; AchoA, anterior choroidal artery; AComA, anterior communicating artery; AICA, anterior inferior cerebellar artery; CCF, carotid cavernous fistulas; CTA, computed tomographic angiography; DED, DERIVO embolization device; Distal arteries: arteries beyond the circle of Willis; DSA, digital subtraction angiography; FD, flow diverter; FRED, Flow-Redirection Endoluminal Device; ICA, internal carotid artery; Ideal Clinical Outcome, modified Ranking Scale ranged from 0 to 2; LA, lenticulostriate artery; MCA, middle cerebral artery; MRA, magnetic resonance angiography; NA, not available; NR, not reported; OA, ophthalmic artery; PCA, posterior cerebral artery; PComA, posterior communicating artery; PED, the pipeline embolization device; PICA, posterior inferior cerebellar artery; RIA, ruptured intracranial aneurysm; SCA, superior cerebellar artery; SFD, the Silk Flow Diverter; small cerebral artery, diameter of distal parent artery ≦ 3.2 mm; UIA, unruptured intracranial aneurysm; VA, vertebral artery; VBA, vertebrobasilar artery.

### Risk-of-Bias and publication bias

Only 5.3% (3/57) of the studies exhibited a low risk-of-bias, while 40.4% (23/57) had a moderate risk, and the remaining had a high risk. Most studies demonstrated a low risk-of-bias in the items of outcome measurement (77.2%, 44/57) and statistical analysis and reporting (66.7%, 38/57). However, 93.0% (53/57) failed to measure important confounders and 45.6% (26/57) did not provide details on participants lost to follow-up. A summary of these assessments is provided in Table S1 (Supplemental Digital Content 2, http://links.lww.com/JS9/B226). There was no evidence of publication bias for any outcome except the overall occlusion/narrowing of covered branches.

### Efficacy of IA treatment with FDSs covering branches

**Figure 1:**
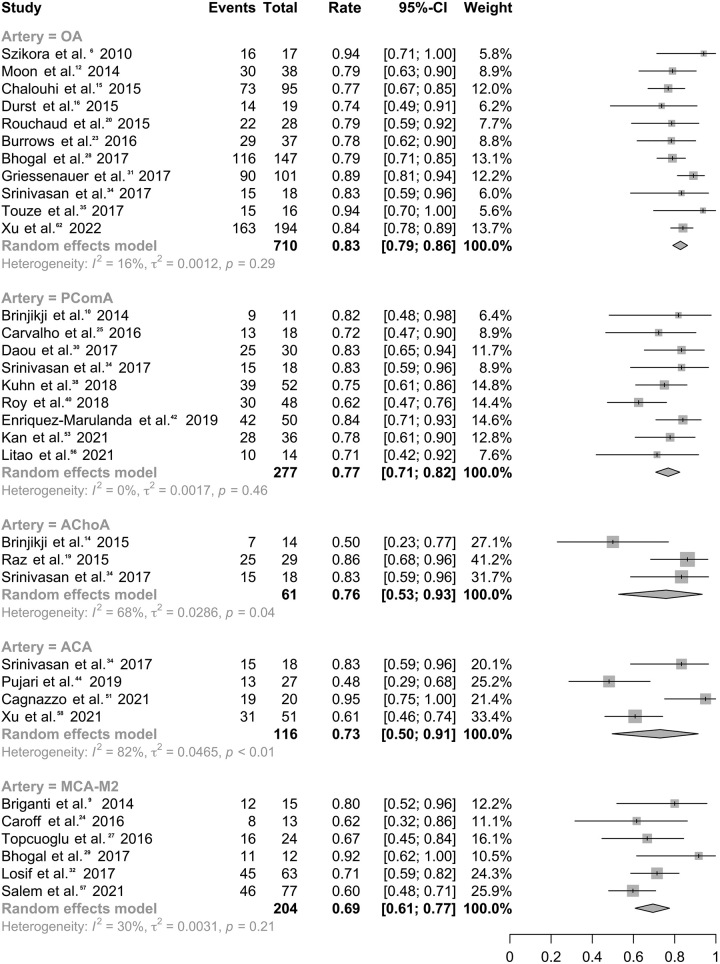
Forest plot for obliteration rates of intracranial aneurysms varied by different flow diverter stents-covering branches.

**Figure 2:**
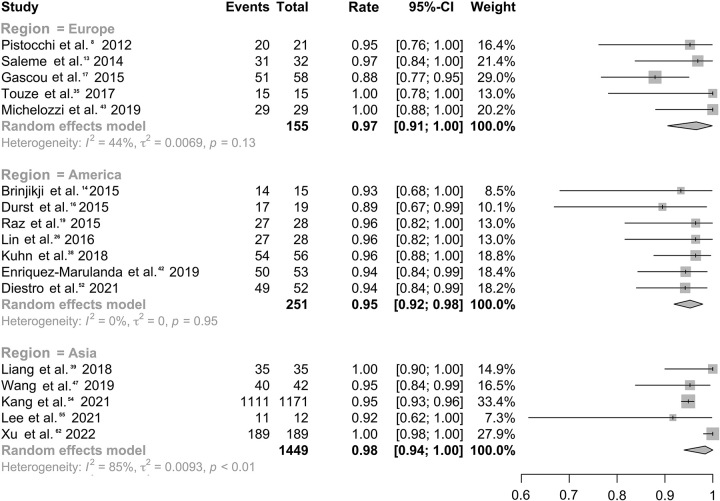
Forest plot for the rate of ideal clinical outcomes varied by studied populations.

Among 50 studies^9–19,22,23,25–35,37–47,49,50,52–63,65^ involving 3100 patients, the overall obliteration rate was 77.5% (95% CI: 74.0–80.9%; *I*^2^, 69.8%; Table S3, Supplemental Digital Content 2, http://links.lww.com/JS9/B226, Figure S2, Supplemental Digital Content 2, http://links.lww.com/JS9/B226). Subgroup analyses indicated a high obliteration rate regardless of the specific branches covered by FDSs (Fig. 1, Figure S3-S4, Supplemental Digital Content 2, http://links.lww.com/JS9/B226) regardless of the specific FDSs placement across the branches of OA (rate, 82.8%; 95% CI: 79.1–86.2%; *I*^2^, 16.1%), PComA (rate, 76.9%; 95% CI: 70.8–82.5%; *I*^2^, 0.0%;), AChoA (rate, 75.8%; 95% CI: 52.8–93.4%; *I*^2^, 67.8%), or ACA (rate, 73.0%; 95% CI: 49.7–91.4%; *I*^2^, 82.1%). Six studies^12,27,30,32,35,60^ reported an occlusion rate of 69.5% (95% CI: 60.8–77.5%; *I*^2^, 30.3%) for IAs when MCA-M2 was covered by FDSs. Two studies^44,51^ reported an occlusion rate of 59.1% (13/22) at the final follow-up when the FDSs covered PICA branches. After excluding the two studies^39,58^, a total of 97.4% of patients (95% CI: 95.5–98.8%; *I*^2^, 54.3%, Table S4, Supplemental Digital Content 2, http://links.lww.com/JS9/B226 and Figure S5, Supplemental Digital Content 2, http://links.lww.com/JS9/B226) showed an ideal clinical outcome, albeit with large heterogeneity^10,11,14,16,17,19,20,22,29,35,38,41,42,45,46,50,55–58,60,65^. Subgroup analysis indicated a relatively lower heterogeneity among studies in America (rate, 95.3%; 95% CI: 91.9–97.9%; *I*^2^, 0.0%) and Europe (rate, 96.5%; 95% CI: 90.6–99.9%; *I*^2^, 44.0%), compared to those in Asia (rate, 98.4%; 95% CI: 94.0–100.0%; *I*^2^, 85.5%, Fig. 2).

### Overall outcomes of complications

After implantation of FDSs covering branches, the overall rate of branch occlusion/narrowing was 26.2% (95% CI: 19.8–33.1%; Figure S6A, Supplemental Digital Content 2, http://links.lww.com/JS9/B226)^9–65^. Due to publication bias (Egger’s test with *P*<0.001), the adjusted rate was 14.5% (95% CI: 8.4–21.8%; Table S5, Supplemental Digital Content 2, http://links.lww.com/JS9/B226 and Figure S6D, Supplemental Digital Content 2, http://links.lww.com/JS9/B226). The pooled rate of branch occlusion-related symptoms (per patient) among 50 included studies^9–30,32–42,44,46,47,49–52,54,56,58–65^ was 1.8% (95% CI: 0.1–3.0%; Table S6, Supplemental Digital Content 2, http://links.lww.com/JS9/B226 and Figure S7, Supplemental Digital Content 2, http://links.lww.com/JS9/B226). Despite the use of subgroup analysis and the omission of several studies, the large heterogeneity remained unresolved. Given the potential for distinct outcomes based on different anatomical characteristics of arteries, we conducted additional subgroup analyses focused to each covered branch, including OA, PComA, AchoA, ACA, MCA-M2, and PICA. The characteristics of these branches are summarized in Table S2 (Supplemental Digital Content 2, http://links.lww.com/JS9/B226).

### Occlusion/narrowing of covered branches

**Figure 3:**
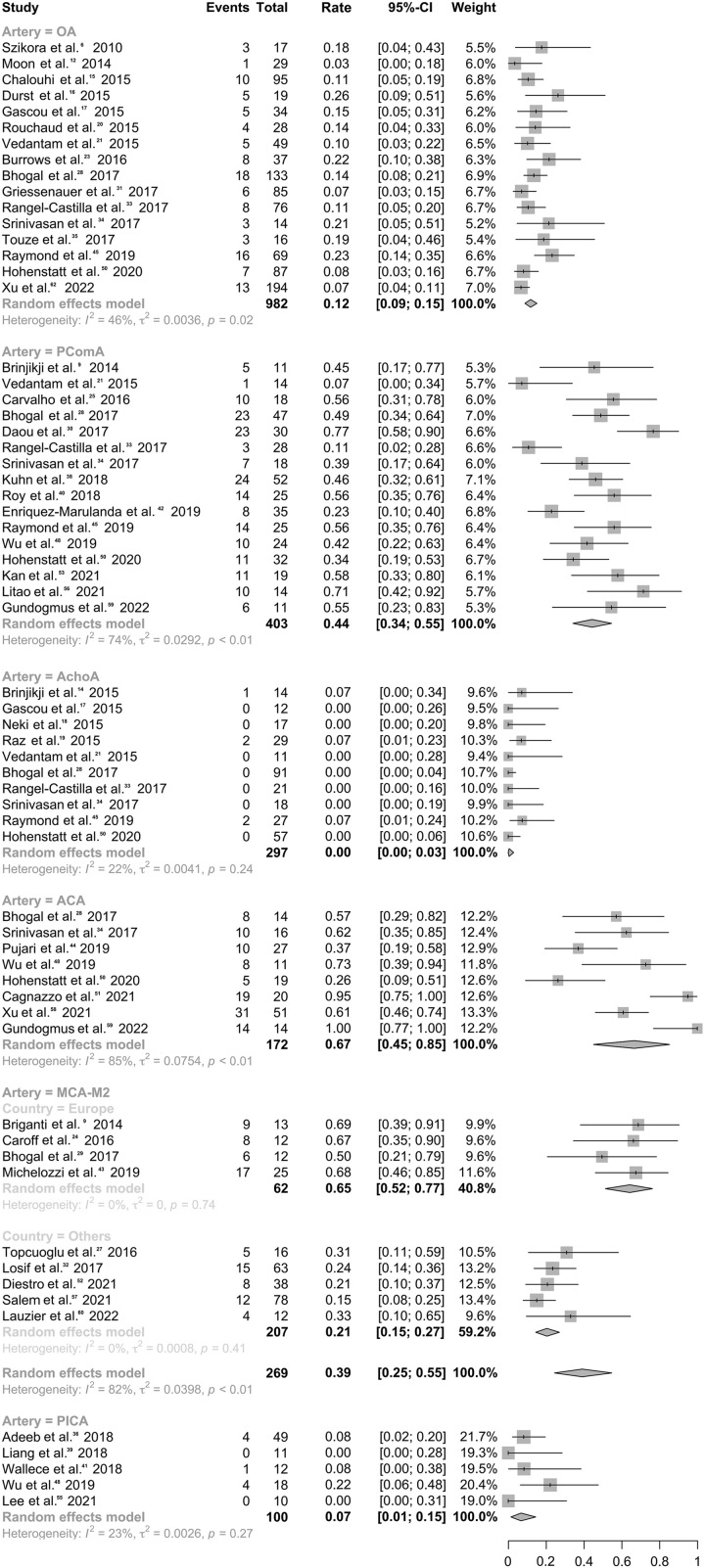
Forest plot for rates of branch occlusion/narrowing varied by different flow diverter stents-covering branches.

The pooled results showed indicated relatively low rates of occlusion/narrowing for OA^9,15,18–20,24,26,31,34,36–38,48,53,65^ (rate, 11.8%; 95% CI: 8.8–15.1%; *I*^2^, 45.9%), AChoA^17,20–22,24,31,36,37,48,53^ (rate, 0.5%; 95% CI: 0.0–2.9%; *I*^2^, 22.5%) and PICA^39,42,47,51,58^ (rate, 6.8%; 95% CI: 1.5–14.5%; *I*^2^, 23.2%). In contrast, higher rates of occlusion or narrowing were observed for PComA^13,24,28,31,33,36,37,41,43,45,48,51,53,56,59,62^ (rate, 44.3%; 95% CI: 34.2–54.6%; *I*^2^, 73.8%), ACA^31,37,47,51,53,54,62,65^ (rate, 66.6%; 95% CI: 45.1–85.3%; *I*^2^, 85.0%) and MCA-M2^12,27,30,32,35,46,55,60,63^ (rate, 39.2%; 95% CI: 24.5–54.7%; *I*^2^, 81.6%). Subgroup analysis reduced the large heterogeneity for the MCA-M2 group, revealing a higher occlusion/narrowing rate in European medical centers^12,27,32,46^ (rate, 64.7%; 95% CI: 51.8–76.7%). Details are presented in Figure 3, Table S5 (Supplemental Digital Content 2, http://links.lww.com/JS9/B226), and Figures S8–S9 (Supplemental Digital Content 2, http://links.lww.com/JS9/B226).

### Branch occlusion-related symptoms

**Figure 4:**
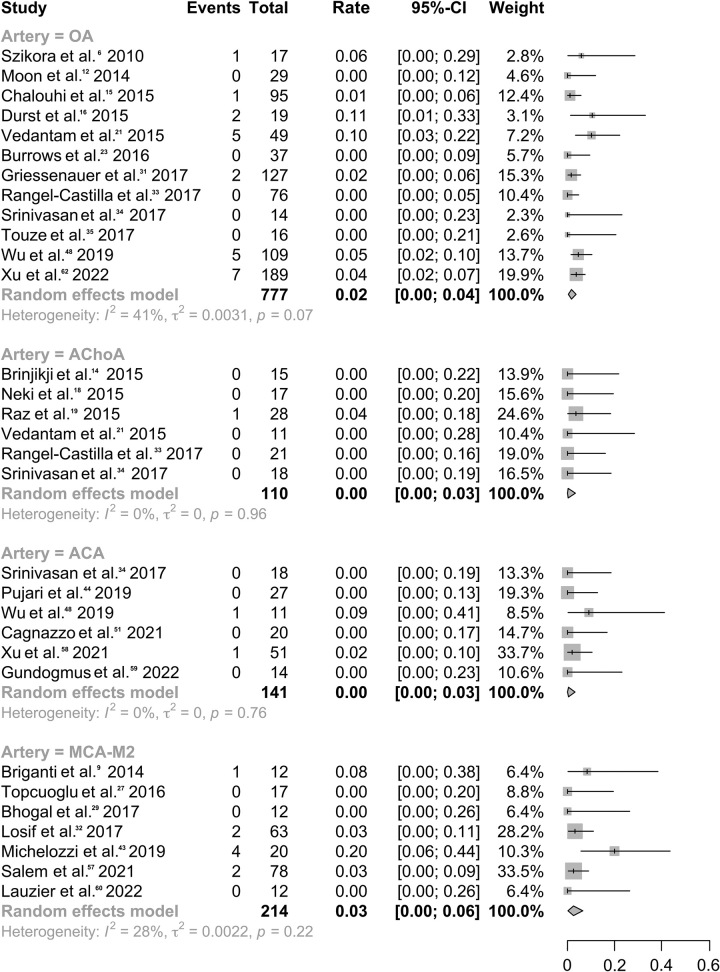
Forest plot for rates of branch occlusion-related symptoms (per patient) varied by different Flow diverter stents-covering branches.

No complications were reported when branches such as PComA^13,24,28,33,36,37,41,51,56,59,62^ or PICA^42,47,51,58^ were covered by FDSs. Additionally, a low risk of symptoms was observed following coverage of AChoA^17,21,22,24,36,37^ (rate, 0.3%; 95% CI: 0–3.3%; *I*^2^, 0.0%) and ACA^37,47,51,54,62,65^ (rate, 0.4%; 95% CI: 0–3.2%; *I*^2^, 0.0%) as shown in Figure 4. New complications arose in 1.6% of patients (95% CI: 0.4–3.6%; *I*^2^, 41.2%) with FDSs covering OA^9,15,18,19,24,26,34,36–38,51,65^, after excluding a heterogeneous study conducted by Rouchaud *et al.* ^23^. The data indicated a relatively higher risk among patients managed with FDSs covering MCA-M2^12,27,30,32,35,46,60,63^ (rate, 2.6%; 95% CI: 0.2–6.5%; *I*^2^, 27.7%). Sensitivity and subgroup analyses identified that the source of heterogeneity was primarily a European study by Caroff *et al.* ^27^. (Table S6, Supplemental Digital Content 2, http://links.lww.com/JS9/B226 and Figures S10–S11, Supplemental Digital Content 2, http://links.lww.com/JS9/B226).

Owing to the limited sample size of the included studies, we directly calculated the rate of complications arising from occluded or narrowed branches. New complications were observed in 33.3% (1/3) of AChoA branches, 28.1% (27/96) of OA branches, and 19.7% (15/76) of MCA-M2 branches after they had been covered by FDSs. In contrast, lower risks were observed in ACA (2/92, 2.2%), PComA (0/110, 0%), and PICA (0/5, 0%) branches.

## Discussion

This study systematically included 57 studies involving 3789 patients with IA treated with FDSs covering intracranial branch arteries. Our synthesis of evidence demonstrated the safety and efficacy of FDSs in managing IAs; 97.4% of patients had ideal clinical outcomes, and 77.5% of IAs were occluded at the last follow-up. However, further research is warranted considering the limited sample size, especially for IAs located distal to the internal carotid artery. Despite higher rates of occlusion/narrowing identified in the covered branches of ACA, PComA, and MCA-M2 compared to those of the OA, PICA, and AChoA, the risk of related complications was acceptable.

Our findings showed a relatively higher risk of occlusion/narrowing for ACA, PComA, and MCA-M2 branches covered by FDSs; however, related clinical complications were rarely observed when covering ACA or PComA branches. This suggests that compensatory cerebral blood flow arises from the contralateral ACA via the anterior communicating artery when the ACA is blocked, and from the posterior cerebral artery when the PComA is blocked. However, ischemic symptoms may occur if predominant arteries are covered and there is insufficient compensation from other branches^66^. The M2 segment of the MCA, due to its relatively distal location, narrower diameter, and thinner walls, struggles to receive sufficient reperfusion when covered by FDSs. A study^67^ on MCA-M2 thrombectomy reported that 58.1% of patients with MCA-M2 occlusion had good outcomes and another study^68^ showed a similar rate of successful MCA-M2 reperfusion, whereas our results showed relatively lower branch occlusion-related complications when the MCA-M2 was covered by FDSs. Because hemodynamics in FDS-covered branches can decrease gradually rather than abruptly in an acute thrombotic event, allowing time for collateral circulation to establish. Infarction of the AChoA can lead to a devastating outcome, AChoA syndrome, manifesting as the triad of hemiparesis, hemianesthesia, and hemianopia^69^. The absence of AChoA infarction was identified as an independent predictor of good outcomes for acute ICA occlusion^70^. However, we found a low risk of AChoA occlusion/narrowing and an unsatisfactory outcome despite cases without considering cases of ICA in-stent occlusion. Furthermore, when FDSs were placed across the OA, only 13.0% were occluded or narrowed, and 2.2% of patients exhibited new clinical symptoms. However, approximately one-third of patients with their OA occluded/narrowed meanwhile experienced new complications. Considering that paraclinoid aneurysms can grow to a large size and have a lower rate of rupture compared to other aneurysms, the source of new symptoms—either from OA occlusion or mass effect caused by coils or IA thrombogenesis—is difficult to pinpoint^71^. Additionally, no new symptoms were observed in patients with a PICA covered by FDSs. We hypothesize that the contralateral PICA or ipsilateral anterior inferior cerebellar artery may compensate for blood flow; however, further investigations with a larger sample size are needed to verify this.

The use of antiplatelets for maintaining branch patency allows for continued protection against further thrombosis induced by FDSs and coils, while minimizing hemorrhagic complications^72^. However, a subgroup analysis could not be conducted due to the variability in antiplatelet regimens among the included studies. Several studies^9,10,12,14,20,22,27,31,35,38,45,52,56,58,60,62,63^ have reported symptomatic thrombotic events of in-stent thrombosis or branch occlusion/narrowing caused by the inappropriate use of antiplatelets; however, several of the included studies did not elaborate on this, so we could not evaluate this association accurately. Additionally, branch occlusion may be observed in patients with in-stent thrombosis; however, these cases were not classified in our study. Consequently, the risks of branch occlusion and related complications may be underestimated.

In IAs management, covering branches during FDSs placement should be avoided to reduce hemodynamic perturbations beyond IAs. If this is not feasible, our results suggest that placing FDSs across the OA, PComA, AChoA, MCA-M2, or ACA is relatively safe. Before the procedure, it is crucial to evaluate the vascular anatomy and hemodynamics to ensure adequate blood supply from collateral circulation. Strict administration of anticoagulants and antiplatelets should be initiated during the perioperative period to prevent thrombotic complications. Considering the occlusion rate of both IA and MCA-M2, caution is advised when placing FDSs at the MCA-M2 bifurcation, especially since microsurgery remains a feasible and safe alternative^73^. There is insufficient evidence on the treatment of IAs using FDSs covering the PICA or other unspecified cerebral arteries, warranting further research to determine their feasibility, effectiveness, and safety.

The risk-of-bias in the included studies mainly arises from issues controlling for confounders and loss to follow-up. Given that FDS is a relatively new innovation, earlier studies employed varied approaches in treating patients with IA, including different perioperative antiplatelet regimens, selection of implanted materials (FDS only, overlapping FDSs, or FDS with coiling), and imaging modalities. This has led to a heterogeneity in reported outcomes. It should be noted that clinicians had avoided obstructing branches while deploying FDSs, due to the risk of thrombosis caused by the decrease of blood flow in FDS-covered vessels. This caution has limited the sample size and design of existing studies. To assess the robustness of our pooled results, we conducted an additional sensitivity analysis among the studies with a sample size ≥20 patients, showing consistent results (Tables S3–S6, Supplemental Digital Content 2, http://links.lww.com/JS9/B226). Randomized clinical trials (RCTs) were valuable but not included in our study because the existing trails of PARAT^74^ or FIAT^75^ did not report on the outcomes of interest. Besides, the randomization was conducted into treatment groups of FDS or coiling with/without stent assistant instead of jailing branches or not jailing branches, and the potential confounders still existed. Even though 12.3% (7/57) of the included studies lost more than 20% of patients in follow-up, there were still a large proportion of included studies (42.1%, 24/57) without loss of participants or reporting details. Another limitation is that the available data restricted further analysis of different types of FDSs and antithrombotic regimens, as well as the use of overlapping or telescopic FDSs, which would also increase the risk of thrombosis due to a higher metal coverage on both IAs and branches. Additionally, significant heterogeneity exists due to variations in surgical techniques and skills among different operators, thus, perspective multicenter cohorts with standardized procedures and adequate follow-up are needed.

Our evaluation comprehensively assessed the occlusion/narrowing of FDS-covered branches and related complications, highlighting substantial evidence gaps in the management of IAs beyond the ICA. Acceptable outcomes were noted when FDSs were placed across the OA, PComA, AChoA, ACA, and MCA-M2. However, neurosurgeons, neurologists, and interventional neuroradiologists should be aware of the risk of complications of branch occlusions when making clinical decisions and should conduct precise preoperative assessments for individualized treatment. The limitations of existing studies underscore the urgent need for multicenter pragmatic trials aimed at long-term, patient-centered outcomes concerning the treatment goals for IA occlusion and fewer complications.

## Ethical approval

Not applicable.

## Consent

Not applicable.

## Sources of funding

This study was supported by Financial Science and Technology Project of Hunan Province, China (422000008), and Central South University Case Database Construction Project for Graduate Students (2020ALK24).

## Author contribution

J.Y.L.: conceptualization, data curation, formal analysis, investigation, methodology, project administration, resources, software, validation, visualization, writing – original draft and review and editing; F.C., N.B.Z.M., Y.X.G.: data curation and resources; Y.F.L. and D.Y.: validation; W.X.J.: investigation, project administration, and validation; J.X.Y.: investigation, project administration, validation, and visualization, writing – original draft and review and editing.

## Conflicts of interests disclosure

There is no competing interest for all of the authors.

## Research registration unique identifying number (UIN)

This study was registered with PROSPERO (CRD42022350339).

## Guarantor

Junxia Yan and Junyu Liu.

## Data availability statement

Data in the manuscript and supplemental files makes use of publicly available data from included studies, and there are no original data for sharing.

## Provenance and peer review

Not commissioned, externally peer-reviewed.
